# 

**Published:** 1851-10

**Authors:** 


### CONTENTS

### OF NO. IV.

---

### ORIGINAL COMMUNICATIONS.

### ESSAYS AND CASES.

Art. I.—Medical Journals and Medical Criticism. By W. S. Chipley, M. D., of Lexington Ky. - - - 277
Art. II.—Remarks on the Use of the Nitrate Silver in Leucorrhea. By Napoleon B. Anderson, M. D., of Louisville, Ky. - - - - - - 294

### REVIEWS.

Art. III—Lectures on the Eruptive Fevers, as now in the course of delivery at St. Thomas's Hospital, London. By George Gregory, M. D., Fellow of the Royal College of Physicians of London; Physician to the Small-Pox and Vaccination Hospital at Highgate; Corresponding Member of the National Institute of Washington, etc. First American edition, with numerous additions and amendments by the Author, comprising his latest views: With notes and an appendix, embodying the most recent opinions on exanthematic pathology; and also statistical tables, and colored plates. By H. D. Bulkley, M. D., Physician of the New York Hospital; Fellow of the New York College of Physicians and Surgeons, etc. etc. 298
Art. IV.—A Practical Treatise on the Diseases and Injuries of the Urinary Bladder, the Prostate Gland, and the Urethra. By S. D. Gross M.D., Professor of Surgery in the University of Louisville, Member of the American Medical Association, author of Elements of Pathological Anatomy, etc. etc. - - - - 314

SELECTIONS FROM FOREIGN AND HOME JOURNALS.

A Case of Rupture of the Intestines, produced by a fall from a horse. - - - - - - - - - 337
Case of Prolapsus Vesicæ. - - - - - - - - 338
Case of Puerperal Convulsions. - - - - - - 340
Case of Stricture of the Bowels. - - - - - - 343
Case of Progressive and Morbid Diminution of the whole body. 347
Urea, its tests and origin. - - - - - - - 350
On the Temperature of Man within the Tropics. - - 353
The Diagnosis of Fevers. - - - - - - - - 354
Two Remarkable Cases of Abstinence. - - - - - 355
Influence of the Poison of Northern Rattle-Snakes on Plants. 356
Demonstrative Midwifery. - - - - - 357
On the use of Collodion in Ingrowing Nail. - - - 358
Quinine in Cholera Infantum. - - - - - 359
A Case of Small-Pox recurring a third time after Vaccination, when it proved fatal. - - - - - - - - 360
Creasote in Diarrhea. - - - - - - - - 360

ORIGINAL INTELLIGENCE.

University of Louisville. - - - - - - - - 361
University of Pennsylvania. - - - - - - - 362
Prize Essays. - - - - - - - - - - 362
Quinine in Cholera Infantum - - - - - - - 362
Hair Blanched by Sudden Fear. - - - - - - 363
Health of Louisville. - - - - - - - - 364
Dr. J. V C. Smith of Boston. - - - - - - 364
Preliminary Lectures. - - - - - - - - 364
The American Medical Association. - - - - - - 366
Dr. Fenner's Southern Reports. - - - - - - 367
Convention of Medical Delegates. - - - - - - 368
Prof. Silliman. - - - - - - - - - 368
Obituary. - - - - - - - - - - 368

